# Bayesian phylogeny analysis of vertebrate serpins illustrates evolutionary conservation of the intron and indels based six groups classification system from lampreys for ∼500 MY

**DOI:** 10.7717/peerj.1026

**Published:** 2015-06-16

**Authors:** Abhishek Kumar

**Affiliations:** Department of Genetics & Molecular Biology in Botany, Institute of Botany, Christian-Albrechts-University at Kiel, Kiel, Germany; Division of Molecular Genetic Epidemiology, German Cancer Research Center (DKFZ), Heidelberg, Germany

**Keywords:** Vertebrates, Serpins, Bayesian phylogeny, Gene structure, Gene duplication, Intron-exon

## Abstract

The serpin superfamily is characterized by proteins that fold into a conserved tertiary structure and exploits a sophisticated and irreversible suicide-mechanism of inhibition. Vertebrate serpins are classified into six groups (V1–V6), based on three independent biological features—genomic organization, diagnostic amino acid sites and rare indels. However, this classification system was based on the limited number of mammalian genomes available. In this study, several non-mammalian genomes are used to validate this classification system using the powerful Bayesian phylogenetic method. This method supports the intron and indel based vertebrate classification and proves that serpins have been maintained from lampreys to humans for about 500 MY. Lampreys have fewer than 10 serpins, which expand into 36 serpins in humans. The two expanding groups V1 and V2 have SERPINB1/SERPINB6 and SERPINA8/SERPIND1 as the ancestral serpins, respectively. Large clusters of serpins are formed by local duplications of these serpins in tetrapod genomes. Interestingly, the ancestral HCII/SERPIND1 locus (nested within PIK4CA) possesses group V4 serpin (A2APL1, homolog of *α*_2_-AP/SERPINF2) of lampreys; hence, pointing to the fact that group V4 might have originated from group V2. Additionally in this study, details of the phylogenetic history and genomic characteristics of vertebrate serpins are revisited.

## Introduction

Serine proteinase inhibitors (serpins) are one of the major regulators of cellular proteolysis. The superfamily of serpins is involved in an array of fundamental biological processes such as blood coagulation, cell differentiation, cell migration, complement activation, embryo implantation, fibrinolysis, angiogenesis, and inflammation, and tumor suppression (Silverman et al., 2001). Serpins usually have a single domain (Pfam ID PF00079 or Interpro ID IPR023795) with a conserved core of ∼350–400 residues. They often possess N- or C-terminal extensions and an overall molecular mass of ∼40–60 kDa. N- and/or O-glycosylations are frequently observed in extracellular serpins (Gettins, 2002; Gettins, Patston & Olson, 1996). The conserved three-dimensional structure of serpins is composed of three *β*-sheets (*β*A–*β*C) and 8–9 *α*-helices (*α*A–*α*I). The hallmark of the serpin inhibitory mechanism is a large-scale conformational change involving the reactive center loop (RCL). The RCL is an exposed flexible loop of about 17–20 residues, which interacts with a target protease (Silverman et al., 2001). RCL acts as a bait imitating the protease substrate, and is cleaved between the positions P1 and P1′ (Silverman et al., 2001).

In metazoans, serpins have undergone divergent evolution over a period of about 650–700 million years (Kumar & Ragg, 2008). A number of phylogenetic studies have been undertaken using sequence analysis of the serpins. Early investigations suggested the establishment of this multigene family through inter- and intra-chromosomal gene duplications. Several gene clusters have arisen, encoding functionally diverse serpin proteins. In metazoans, serpins display highly variant exon-intron patterns are strongly conserved within some taxa. Gene architecture and other rare genetic characters singularize a robust basis for classifying vertebrate serpins. Based on number, positions, and phases of introns, serpins have been classified into six groups (V1–V6). Vertebrate serpin genes with equivalent gene structures often tend to be organized in clusters (Benarafa & Remold-O’Donnell, 2005). However, close physical linkage cannot always be established. Interestingly, none of the 24-intron positions that have been mapped to the core domain of vertebrate serpins is shared by all of the six gene groups. Nevertheless, characteristic amino acid indels provide further cues to unravel the phylogenetic relationship (Ragg et al., 2001). Previous analyses were performed using limited vertebrate serpins, and mainly focused on human and mouse data. Currently, several non-mammalian vertebrate genomes are known. Hence, combining these genomes for validation of intron-encoded vertebrate serpin classification is possible. In addition, different phylogenetic methods have been applied to vertebrate serpin classification such as maximum-likelihood (ML) and Neighbor-joining (NJ) (Atchley et al., 2001). In the last decade, Bayesian Markov chain Monte Carlo (MCMC) has been enthusiastically corroborated as the state-of-the-art method for phylogenetic reconstruction and was largely driven by the rapid and widespread adoption of MrBayes suite (Ronquist & Huelsenbeck, 2003). This method was recently tested for urochordate serpin classification (Kumar & Bhandari, 2014). Heretofore, there is no report on the use of this phylogenetic method for large-scale analysis of vertebrate serpins. Herein, Bayesian method was employed along with several non-mammalian genomes for reconstructing vertebrate serpin classification system. This study reveals that Bayesian phylogenic method supports the intron-coded vertebrate serpin classification system.

Moreover, this classification system is conserved from lampreys to human with a few new introns being created in groups V2, V4 and V6 in the serpin core domains of selected ray-finned fishes. Furthermore, different properties of these six serpin groups were also summarized.

## Materials and Methods

### Collection of serpins from selected genomes

Serpin sequences of selected vertebrates (Table 1) were obtained from the Ensembl database (release 72, June 2013) using the BLAST suite (Altschul et al., 1997). Details of identified serpins and comprehensive alignments are provided (Kumar, 2010).

**Table 1:** Vertebrate genomes used during this study.

*Genome*	Major database used	References
*Homo sapiens*	http://www.ncbi.nlm.nih.gov/genome/guide/human/	(Venter et al., 2001)
*Mus musculus*	http://www.ncbi.nlm.nih.gov/genome/guide/mouse/	(Mouse Genome Sequencing Consortium et al., 2002)
*Rattus norvegicus*	http://www.ncbi.nlm.nih.gov/genome/guide/rat/	(Gibbs et al., 2004)
*Gallus gallus*	http://www.ncbi.nlm.nih.gov/genome/guide/chicken/	(Hillier et al., 2004)
*Xenopus tropicalis*	http://genome.jgi-psf.org/Xentr4/Xentr4.home.html	(Hellsten et al., 2010)
*Fugu rubripes*	http://genome.jgi-psf.org/Takru4/Takru4.home.html	(Aparicio et al., 2002)
*Tetraodon nigroviridis*	http://www.genoscope.cns.fr/externe/tetranew/	(Jaillon et al., 2004)
*Danio rerio*	http://www.ensembl.org/Danio_rerio/index.html	(Birney et al., 2006)
*Petromyzon marinus*	http://www.ensembl.org/Petromyzon_marinus/Info/Index	(Smith et al., 2013)

### Gene structure prediction of serpins

To ensure accuracy, gene structure prediction within the Ensembl (Flicek et al., 2013) was taken and combined with predictions of AUGUSTUS gene prediction tool (Stanke & Morgenstern, 2005). Mature human *α*_1_-antitrypsin was used as the standard sequence for intron position mapping and numbering of intron positions, followed by suffixes a–c for their locations as reported previously (Kumar & Ragg, 2008).

### Protein sequence alignment

Protein alignments of different vertebrate serpins were created by MUSCLE tool (Edgar, 2004) using default parameters.

### Selection of substitution model

Upon evaluation of different amino acid substitution models for this alignment dataset using MEGA5 software suite (Tamura et al., 2011), it turned out that the WAG + G model was the best fit (Table S1).

### Bayesian phylogenetic analysis

To infer the evolutionary history of serpins, the Bayesian phylogenetic tree was constructed using the MrBayes 3.2 suite (Ronquist & Huelsenbeck, 2003) with the following parameters: 5 generations, until average standard deviation of split frequencies was lower than 0.0098, 25% burn-in-period, WAG + G matrix-based model. Nve_Spn1 from starlet sea anemone (*Nematostella vectensis*) was used as the outgroup.

### Estimation of genome size

Selected vertebrate genome sizes were calculated using the animal genome size database (Gregory, 2014).

## Results and Discussion

### Bayesian phylogeny classifies serpins into six groups V1–V6

**Figure 1:**
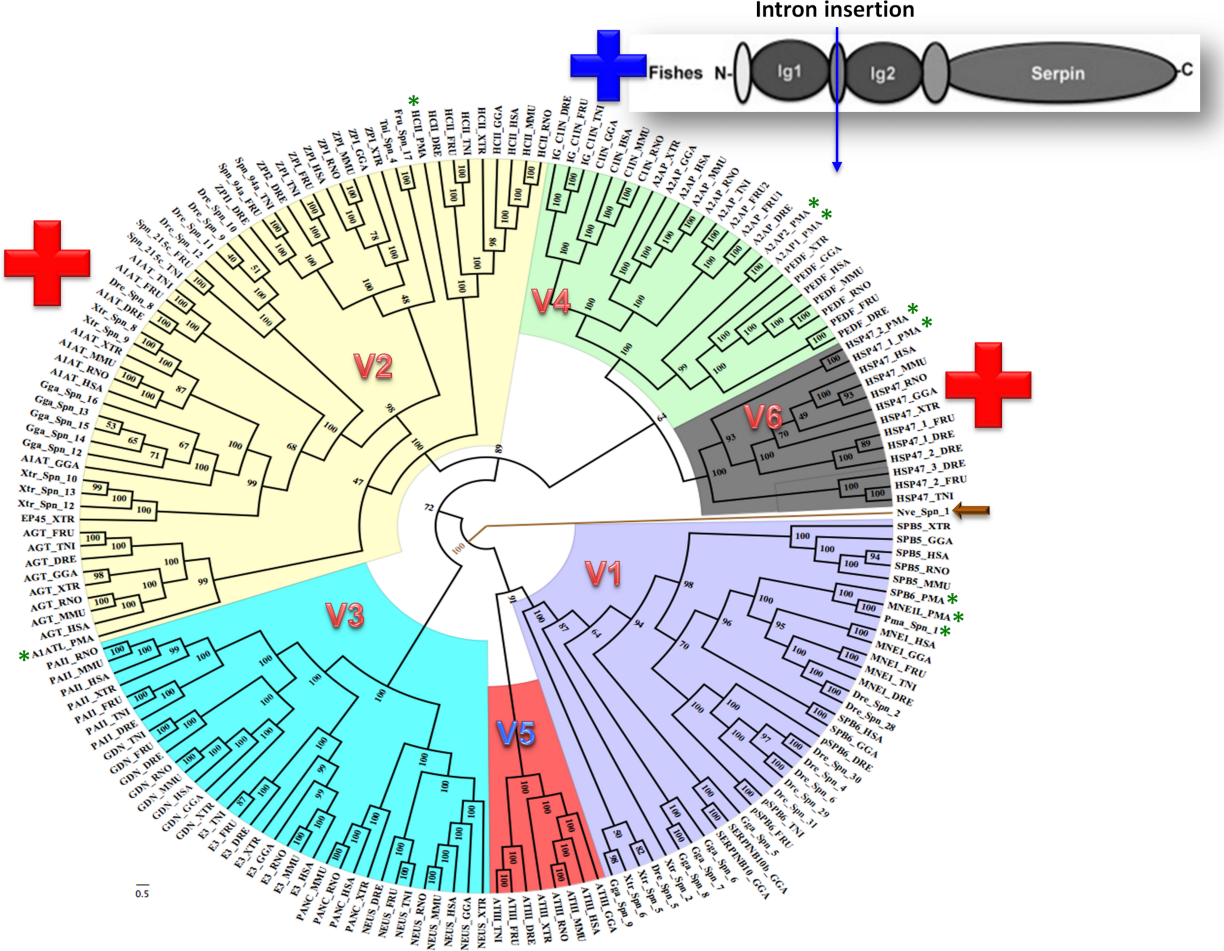
Bayesian phylogenetic history of vertebrate serpins reveals that exon-intron and rare indel based classification system is retained over period of 500 MY with conserved patterns from early diverging lampreys. Novel introns are inserted in groups V2 and V6 serpins in core domains are marked by red + while introns inserted in additional Ig domains of fish-specific C1 inhibitors is shown with a blue +. A sea anemone serpin (Nve_Spn1) is the out-group for this phylogenetic analysis as marked by an arrow. Lamprey serpins are marked by green stars. HSP47 has two isoforms in lamprey named as HSP47_1_PMA and HSP47_2_PMA. DRE, Danio rerio; H SA, Homo sapiens; GGA, Gallus gallus; MMU, Mus musculus; PMA, *Petromyzon marinus*; RNO, *Rattus norvegicus*; TRU, *Takifugu rubripes*; TNI, *Tetraodon nigroviridis*; XTR, *Xenopus tropicalis*. *p*-paralog of a gene.

**Figure 2:**
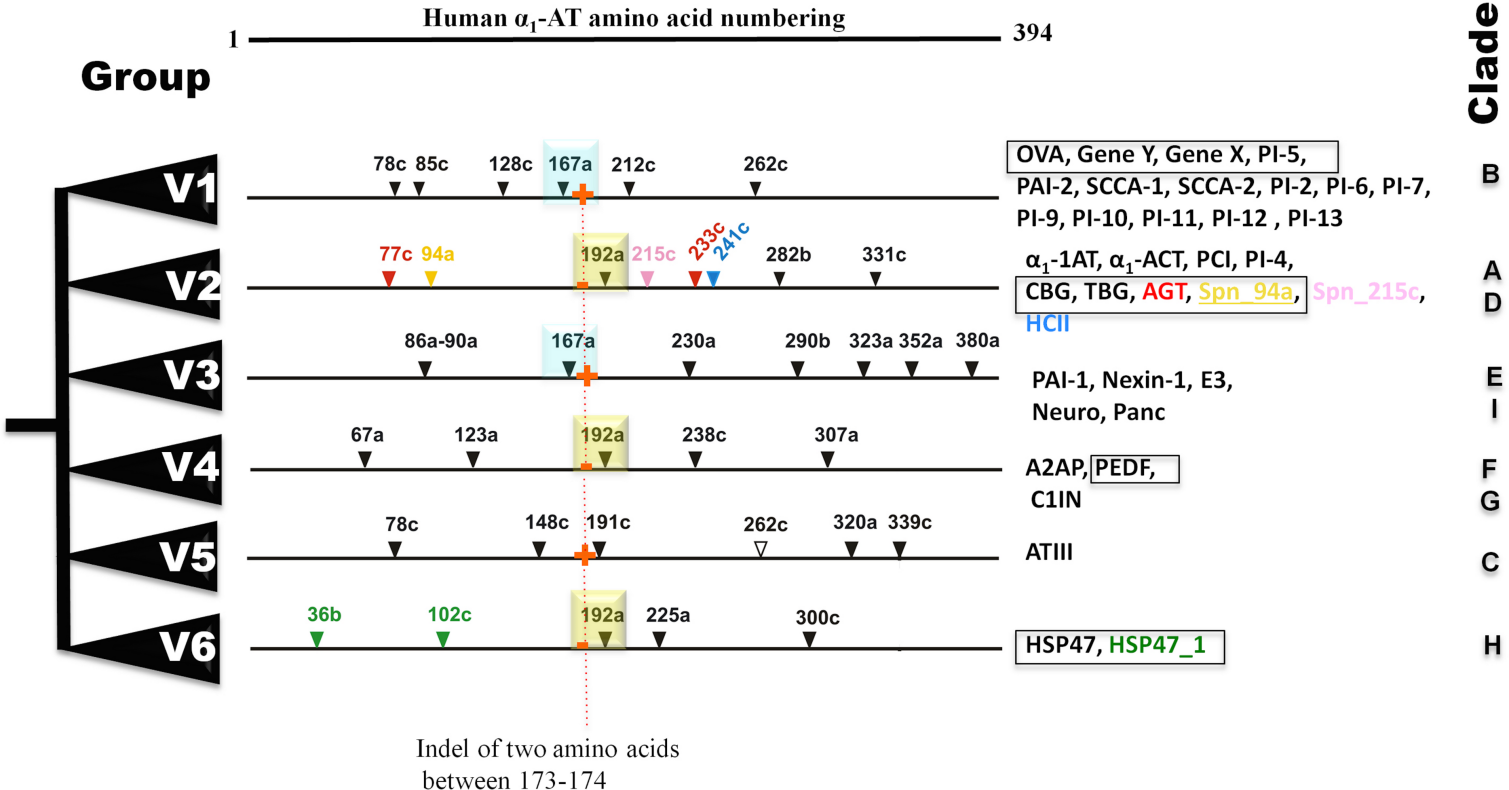
Summary of six groups (V1–V6) classification system of vertebrate serpins, based on introns and rare indels. Conserved intron positions are shown in cyan and yellow boxes for positions 167a and 192a, respectively. Fish-specific introns are inserted in selected serpins are illustrated in different colors. Non-inhibitory serpins are shown in square boxes. Presence and absesnce of sequence indel of two amino acid between positions 173–174 are marked in by red + and − signs. OVA, Ovalbumin; Gene Y, Chicken gene Y protein; Gene X, Chicken gene X protein; PAI, Plasminogen activator inhibitor; SCCA, Squamous cell carcinoma antigen; *α*_1_-AT, *α*_1_-antitrypsin; *α*_1_-ACT, *α*_1_-antichymotrypsin; CBG, -Corticosteroid-binding globulin; TBG, Thyroxine-binding globulin; HCII, Heparin cofactor II; PCI, Protein C inhibitor; AGT, Angiotensinogen; E3, SerpinE3; Neuro, Neuroserpin; Panc, Pancpin; A2AP, *α*_2_-Antiplasmin; PEDF, Pigment epithelium derived factor; C1IN, C1-Inhibitor; ATIII, Antithrombin III; HSP47, Heat shock protein 47kDa.

Bayesian phylogenetic analysis reveals six groups of vertebrate serpins as depicted in different colors (Fig. 1). Posterior probability values are marked, with the lowest being 47, because several paralogs of group V2 serpins are known in tetrapod genomes (Fig. 1). Sea anemone serpin (Nve_Spn1) is the out-group for this phylogenetic analysis (marked by a brown arrow). This clustering matches with intron-indel based vertebrate serpins classification system of six groups (V1–V6) as illustrated in Fig. 2. Lampreys have only eight serpins as evident from BLAST analysis against sea lamprey (*Petromyzon marinus*) genome and cDNA of European river lamprey, *Lampetra fluviatilis* in the Genbank (Table 2). These serpins of lampreys are only distributed into four groups (marked by green stars in Fig. 2). I will describe and discuss each of these groups in next sections.

**Table 2:** Summary of serpins in two lampreys namely, sea lamprey (*Petromyzon marinus*) and European river lamprey (*Lampetra fluviatilis*).

Name given	Ensembl Accession id	Serpin name	Group	Clade	RCL with P1–P1′	Ortholog in *L. fluviatilis* Genbank ID
Pma-Spn-1	ENSPMAG00000001963	SERPINB1-like	V1	B	GTEAAAATAAIVMMR–CARMG	
MNE1L_PMA/Pma-Spn-2	ENSPMAG00000009027	MNEI1-like/SERPINB1-like	V1	B	GTEAAAATAVTMKLR–CAMPT	
SPB6_PMA (Pma-Spn-3)	ENSPMAG00000009040	SPB6/SERPINB6-like	V1	B	GTEAAAATAISVMLM–CAMPT	
A1ATL_PMA (Pma-Spn-4)	ENSPMAG00000006108	A1AT-like, angiotensinogen, SERPINA8	V2	A	GTEAKAETVVGIMPI–SMPPT	CAV16871.1/CAV29466.1
A1ATL_PMA (Pma-Spn-5)	ENSPMAG00000008131	Heparin cofactor II/SERPIND1	V2	D	GSEAAAVTTVGFTPL–TSHNR	CAX18777.1/AIA57696.1
A2APL1_PMA (Pma-Spn-6)	ENSPMAG00000008124	Alpha-2-antiplasmin-like 1	V4	F	GVKATAATGIMISLM–SVQHS	CAX18777.1/CAX18778.1
A2APL2_PMA (Pma-Spn-7)	ENSPMAG00000002992	Alpha-2-antiplasmin-like 2, A2APL2_PMA	V4	F	GAEAAAVTGVFLSRT–NPIYP	AIE16052.1/AIE16053.1
HSP47_PMA (Pma-Spn-8)	ENSPMAG00000007485	HSP47/SERPINH1	V6	H	GEEYDMSVHGHPDM–RNPHL	

### Group V1 has several clade B members expanded from fishes to human

Group V1 serpins has been defined by a gene structure depicting five introns at positions—78c, 128c, 167a, 212c, and 262c, in their coding region (Fig. 2). An additional intron at the position 85c is found in some group V1 members and the presence and absence of this intron, defines sub-groups V1a and V1b, respectively. Group V1 is multi-membered, consisting of ovalbumin-like serpins that are involved in different physiological roles and often called as ov-serpins (Benarafa & Remold-O’Donnell, 2005). These serpins belong to clade B under clade-based classification system of serpins (Silverman et al., 2001). They are usually inhibitors of serine or cysteine proteases (cross-class inhibition), but some of them are non-inhibitory members (e.g., maspin/SERPINB5). They are mostly intracellular since they lack N-terminal signal peptide with few exceptions (Benarafa & Remold-O’Donnell, 2005; Izuhara et al., 2008; Kaiserman & Bird, 2005). They are also deprived of the C-terminal extensions (Benarafa & Remold-O’Donnell, 2005; Izuhara et al., 2008; Kaiserman & Bird, 2005). These serpins are localized in two clusters in the human genome. Human chromosome 6p25 region harbors three genes—SERPINB1, SERPINB6, and SERPINB9 as the first cluster while, the remaining genes namely, SERPINB2, SERPINB3, SERPINB4, SERPINB5, SERPINB7, SERPINB8, SERPINB10, SERPINB11, SERPINB12, and SERPINB13 are located in the 18q21 region (Benarafa & Remold-O’Donnell, 2005; Izuhara et al., 2008; Kaiserman & Bird, 2005).

This cluster originated by duplication of SERPINB1/MNEI1-like gene (Benarafa & Remold-O’Donnell, 2005). In contrast, the chicken has only one cluster on the chromosome 2q. Therefore, it is corroborated that there was a split after mammal/bird divergence at about 310 MY (Benarafa & Remold-O’Donnell, 2005; Izuhara et al., 2008; Kaiserman & Bird, 2005). Similar genomic organization of group V1 was also found in frogs and fishes. In addition to this syntenic organization, fishes possess some paralogous clusters of group V1 serpins while an additional cluster containing two serpins adjacent to the conserved orthologous cluster is found in frogs. The serpins SERPINB1/SERPINB6 of group V1 are probably the ancestors of all group V1 serpins, as these genes are found in lampreys (Table 2) and other fishes and are also conserved across other vertebrate taxa (Kumar, 2010). The group V1 serpins may be classified into sub-groups V1a and V1b, since these differ by one intron. It has been argued that a serpin gene of group V1b (7 exons) is the ancestor of group V1a (8 exons) that has emerged in birds after the divergence of frogs (Benarafa & Remold-O’Donnell, 2005; Izuhara et al., 2008; Kaiserman & Bird, 2005). The first argument coincides with the current data and corroborates that groups V1a serpins are derived from 7-exon genes (such as MNEI/SPB6). However, the argument that 8-exon genes first arose in chickens is not in agreement with the current data, since Xtr-Spn-5 in *X. tropicalis* and pSPB6 in *T. nigroviridis,* are group V1a members with the 8 exons/7 introns architecture (Kumar, 2010). Therefore, it is proposed that group V1b is ancestral to all group V1 serpins and group V1a is suggested to have arisen independently several times in different vertebrates from fishes to mammals. The ancestor of group V1 serpins appears to have been generated during the emergence of vertebrates. The oldest group V1 serpins are SPB1/SPB6 orthologs, which are present in lamprey (Table 2). A recent study claimed an ancestor of serpinB6 to be present in urochordates (Benarafa & Remold-O’Donnell, 2005). However, another study depicted six different groups of urochordate serpins, based on intron-encoded classification system, which markedly differs from vertebrates six groups (Kumar & Bhandari, 2014).

### Group V2 possesses *α*_**1**_-antitrypsin-like serpins, angiotensinogen (clade A) and heparin cofactor II (clade D)

Group V2 serpins are characterized by three introns at homologous positions—192a, 282b, and 331c (Fig. 2) in their coding regions, and most of the members have an intron mapping to the untranslated regions. This group is multi-membered, composed of *α*_1_-antitrypsin-like serpins that are involved in different physiological roles, including inhibitors, like *α*_1_-antitrypsin or antichymotrypsin as well as non-inhibitory members, like angiotensinogen (AGT/SERPINA8 Kumar, Sarde & Bhandari, 2014).

Gene structures of heparin cofactor II (HCII/SERPIND1) are variable in fishes with a novel intron gain at the position 241c, but this gene is nested in the large intron of phosphatidylinositol 4-Kinase (PIK4CA) gene for ∼500 MYA (Kumar et al., 2014a). Human HCII/SERPIND1 has 985 germline variants, identified from 1,092 human genomes. This includes 37 statistically deleterious missense variants (Kumar et al., 2014a).

Recently, it was reported that the gene structures of AGT from selected ray-finned fishes varied in exons I and II, with insertions of two novel introns in the core domain for ray-finned fishes at positions 77c and 233c, respectively (Kumar, Sarde & Bhandari, 2014). It was also reported that the AGT loci is conserved from lampreys to human and was estimated to be older than 500 MY (Kumar, Sarde & Bhandari, 2014). Interestingly, the RCL of AGT protein is inhibitory in lampreys and evolved to become non-inhibitory in human over a period of 500 MY (Kumar, Sarde & Bhandari, 2014). Additionally, 690 AGT variants were detected by analyzing 1,092 human genomes with the top three variation classes belonging to single nucleotide polymorphisms (SNPs, 89.7%), somatic SNVs (5.2%) and deletion (2.9%) (Kumar, Sarde & Bhandari, 2014). Furthermore, 121 missense variants of AGT (including 32 statistically deleterious variants) were deciphered (Kumar, Sarde & Bhandari, 2014).

From fishes to humans, group V2 comprises of multiple paralogs of *α*_1_-antitrypsin-like genes. Genuine orthologs of AGT/SERPINA8 and HCII SERPIND1 were identified from lampreys to humans, using synteny and signature sequences. Concerning the other genes of group V2; since in most tetrapod genomes, *α*_1_-antitrypsin-like gene clusters are derived from recent duplication events, which results in proteins with high sequence similarities, one-to-one orthology allocation proved to be difficult. This poses notorious challenges in detection of orthologs within this cluster and often leads into problems in generating phylogenetic trees (Fig. 1).

In the cluster of *α*_1_-antitrypsin-like genes, the protein Z-dependent protease inhibitor (ZPI/SERPINA10) is localized at the end of the cluster with other conserved marker genes (Kumar, 2010). This assisted in the detection of ZPI/SERPINA10 orthologs using synteny analysis. Recently published report on the serpins of channel catfish, *Ictalurus punctatus* (Li et al., 2015) also supported this finding.

Additionally, two group V2 serpins are found only in ray-finned fishes. The first serpin was detected with a novel intron at position 94a and hence it is named as the Spn_94a gene fishes, which have conserved in the same genomic organization in these fishes. This corroborates that fish specific ortholog Spn_94a shows sequence similarity with ZPI/SERPINA10 gene and hence it is paralog of ZPI/SERPINA10. Similarly, *Fugu* and *T. nigroviridis* possess the second fish-specific group V2 gene with an additional intron at the position 215c (Spn_215c), which indicates that they are orthologs (Kumar, 2010). The origin of these genes, however, is unclear.

No orthologs of the hormone binding serpins (corticosteroid-binding globulin [CBG/SERPINA6] and thyroxine-binding globulin [TBG/SERPINA6]) were detected in non-mammalian vertebrates. In short, the conserved set of group V2 comprises only orthologs of AGT/SERPINA8 (Kumar, Sarde & Bhandari, 2014) and HCII/SERPIND1 (Kumar et al., 2014a). In contrast, some fish-specific group V2 genes and the *α*_1_-antitrypsin-like genes are differentially expanded in vertebrates, particularly in mammalian lineages, such as rodents (Forsyth, Horvath & Coughlin, 2003) and cattle (Pelissier et al., 2008). The expansion of group V2 members should be further explored by analyzing marsupials and Platypus, which branched out early in mammalian evolution. The presence of group V2 members in the lamprey genome suggests that this group originated during emergence of vertebrates (Table 2). Further investigation of group V2 members in the hagfish genome and several lamprey genomes will shed more light on this issue.

### Group V3 is composed of 5 members, which belongs to two clades E and I

Group V3 serpins have seven introns at the positions - 86a/88a/90a, 167a, 230a, 290b, 323a, 352a and 380a in their coding regions (Fig. 2). The exact location of the first intron is uncertain in different group V3 serpins, due to alignment ambiguities. Group V3 has five inhibitory serpins, which are involved in different physiological processes namely, plasminogen activator inhibitor 1 (PAI1/SERPINE1), glia derived nexin (GDN/SERPINE2/), SERPINE3, neuroserpin/SERPINI1, and pancpin/SERPINI2. PAI1/SERPINE1 is conserved in vertebrates, depicting 38–80% sequence identity and 59–95% sequence similarity at the amino acid level with human PAI1/SERPINE1. The inhibitory RCL region is conserved and consists of R–M at the P1–P1′. GDN/SERPINE2 is also highly conserved in vertebrates and it shows 51–84% sequence identity and 70–93% sequence similarity with human GDN/SERPINE2. The helix-D region is highly conserved among GDN/SERPINE2 orthologs of different vertebrates and an N-glycosylation site (positions 163-165) is also conserved. The inhibitory RCL region is also strongly conserved. SERPINE3 is maintained in vertebrates, show 27–64% sequence identity, and 37–74% sequence similarity on the amino acid level with human serpinE3. The inhibitory RCL of SERPINE3 is conserved with a cluster of hydrophobic amino acids preceding the presumptive P1 position.

The neuroserpin/SERPINI1 is highly conserved in vertebrates, and the protein shows 47–81% sequence identity and 65–95% sequence similarity with the human ortholog. The inhibitory RCL region of neuroserpin/SERPINI1 always contains an arginine at the position P1. An N-glycosylation signal at residues 163-165, and a C-terminal extension that has have been shown to direct neuroserpin to the regulated secretory pathway (Ishigami et al., 2007) are strongly conserved.

Discriminatory data at the genomic, gene and protein levels offered comprehensive insight into the phylogenetic history of neuroserpin/SERPINI1 (Kumar & Ragg, 2008). Synteny analysis proved to be very instrumental in this respect, demonstrating that rare genomic characters can provide very useful information for decoding of links between protein families with intricate evolutionary history. The strongly conserved syntenic association of PDCD10 and neuroserpin/SERPINI1 orthologs during diversification of deuterostomes is unraveled (Kumar & Ragg, 2008). These head-to-head oriented genes (Neuroserpin/SERPINI1 and PDCD10) have common bi-directional and asymmetrical promoter region inserted within the ∼0.9 kb intergenic region (Chen et al., 2007). Requirements of common regulatory units could have driven the preservation of this linkage. In the era of next-generation genome sequencing, the rapidly accumulating genome sequences will certainly continue to provide further discriminatory markers, such as codon usage dichotomy (Krem & Di Cera, 2003), in order to facilitate robust classification of other metazoan serpins.

Some serpins have signals for sub-cellular localization at the C-terminal end and they have been involved in the secretory pathway. It is not just limited to vertebrate serpins and several examples exist in invertebrates such as in some urochordate serpins (Kumar & Bhandari, 2014). Using these signals, ancestral orthologs of neuroserpin/SERPINI1 were deciphered (Kumar & Ragg, 2008). A C-terminal KDEL-like motif deters the secretion of soluble endoplasmic reticulum (ER)—resident proteins (Lewis, Sweet & Pelham, 1990; Raykhel et al., 2007; Semenza et al., 1990). There are 24 possible variants of ER retention signals listed as a PROSITE motif—[KRHQSA]-[DENQ]-E-L in the PROSITE database (Sigrist et al., 2013). In addition, there are some ER retention signals that do not fit into the classical PROSITE motif (Raykhel et al., 2007). These ER retention signals are present across eukaryotic genomes such as in the BEM46 protein of *Neurospora crassa* (Kumar, Kollath-Leiss & Kempken, 2013). In early diverging deuterostomia, the neuroserpin orthologs in *Strongylocentrotus* (Spu-spn-1), lancelet (Bfl-Spn-1) and sea anemone (Nve-Spn-1) have HEEL, KDEL, and SDEL at their C-terminal ends, respectively. These are variants of the PROSITE motif for ER retention/retrieval signal. In contrast, the C-terminal end of tetrapod neuroserpin is HDFEEL. In HeLa cells that express three different KDEL receptors with overlapping, but differential passenger specificities, the “FEEL” sub-sequence targets attached passenger proteins primarily to the Golgi, though one-fourth of cells depict ER localization (Raykhel et al., 2007). However, in transfected COS cells, intracellular neuroserpin localizes either to the ER or to Golgi (Ishigami et al., 2007). Conversely, in cells with a regulated secretory pathway, neuroserpin/SERPINI1 resides in large dense core vesicles, which is assisted by a C-terminal extension encompassing the last 13 amino acids (ETMNTSGHDFEEL) including the FEEL sequence (Ishigami et al., 2007). Collectively, these data suggest that in the neuroserpin orthologs from deep-branching metazoans, a two amino acid insertion ‘FE’ in combination with additional residues constitutes a modified sorting signal, which attributes a specialized subcellular localization. Surveillance of the secretory pathway routes by serpins is an ancient and conserved trait in eukaryotes as indicated by the putative neuroserpin ortholog present in the sea anemone genome. It will be interesting to investigate experimentally, whether the C-terminal extensions of neuroserpin orthologs from fishes are functional and mediate differential localization in a fashion similar to mammalian neuroserpin.

Due to variations in their RSL region, ER-localized serpins may work differently in the secretory pathway. Neuroserpin from vertebrates inhibits tissue-type plasminogen activator (tPA) *in vitro*, using the Arg residue at the P1 position in the RSL region (Osterwalder et al., 1998). The cleavage site of Bfl-spn-1 is preceded by the dipeptide motif Lys-Arg (KR), a distinct feature for substrates and inhibitors of proprotein convertases (PCs). Similar features were reported for Bla-Spn-1 from *B. lanceolatum* as well (Bentele et al., 2006). Since the serpins Bfl-spn-1 (*B. floridae*), Spu-spn-1 (sea urchin) and Nve-Spn-1 (sea anemone) also possess the Lys-Arg (KR) dipeptide motif. Thus, similar physiological role of these serpins can be expected. Status of the neuroserpin ortholog in the arthropod lineage is recently becoming clear. Examples of classical ER targeting signal (HDEL) possessing serpins were found in *D. melanogaster* as Spn4, which is a furin inhibitor (Oley, Letzel & Ragg, 2004; Osterwalder et al., 2004; Richer et al., 2004) and its homologous gene in *Anopheles gambiae* as *Spn10* (Danielli, Kafatos & Loukeris, 2003). Recently, the crystal structure of fly Spn4 was determined and this serpin exhibits structural properties as of human neuroserpin/SERPINI1 (Ellisdon et al., 2014), which provided first evidences of the orthologous nature of these serpins.

The pancpin/SERPINI2 gene is localized in close proximity to the neuroserpin gene. Pancpin also possesses a C-terminal extension and indels like neuroserpin, suggesting its close relatedness to these proteins. Pancpin/SERPINI2 orthologs are found only in mammals and in *Xenopus*, showing 49–76% sequence identity, and 68–88% sequence similarity at the amino acid level. The C-terminal end is strongly conserved (Kumar, 2010). Absence of pancpin/SERPINI2 in fishes hints that the pancpin gene may have originated by tandem duplication of neuroserpin after separation of tetrapods from the fish lineage.

In the human genome, the other group V3 members such as PAI1/SERPINE1 (chromosome 3), GDN/SERPINE2 (chromosome 2) and SERPINE3 (chromosome 13) are present at various genomic locations. This suggests that they originated at independent loci in the vertebrates.

### Group V4 has three serpins—two in the clade F and one in the clade G; surprisingly, fishes have C1IN with two immunoglobulin domains

Group V4 of vertebrate serpins have a gene structure consisting a conserved set of five introns at positions 67a, 123a, 192a, 238c, and 307a in the coding regions (Fig. 2). In mammals, group V4 serpins consists of three genes—pigment epithelium derived factor (PEDF/SERPINF1), *α*_2_-antiplasmin (*α*_2_-AP/SERPINF2) and C1 inhibitor (C1IN/SERPING1). Group V4 serpins are involved in very different physiological functions. PEDF is a non-inhibitory serpin that possesses neuroprotective and antiangiogenic functions (Sawant et al., 2004; Steele et al., 1993; Tombran-Tink, 2005). *α*_2_-antiplasmin is an inhibitor of plasmin and its fibrin bound form is a major regulator of blood clot lysis (Coughlin, 2005). C1 inhibitor (C1IN/SERPING1) is a multi-functional serpin, which operates by inactivating various serine proteases in different plasmatic cascades including the complement (classical pathway—C1r and C1s; as well as lectin pathways—MASP1 and MASP2), contact (Factor XII and kallikrein), coagulation (Factor XI and thrombin) and fibrinolytic (tPA and plasmin) systems (Davis, Cai & Liu, 2007; Davis, Lu & Mejia, 2010). Fishes have C1IN/SERPING1 with two immunoglobulin-like domains attached at the *N*-terminal region (Fig. 4). The RCL regions of C1IN/SERPING1 have variations at the positions P2 and P1′ from fishes and tetrapods Gene structures of C1IN/SERPING1 from selected ray-finned fishes varied in the Ig domain region with the insertion of a novel intron splitting exon Im2 into Im2a and Im2b (Kumar et al., 2014b). Kumar & Bhandari (2014) depicted that C1IN/SERPING1 gene has remained on the same locus for ∼450 MY in 52 vertebrates, but it is missing in frogs and lampreys (Kumar et al., 2014b).

**Figure 3:**
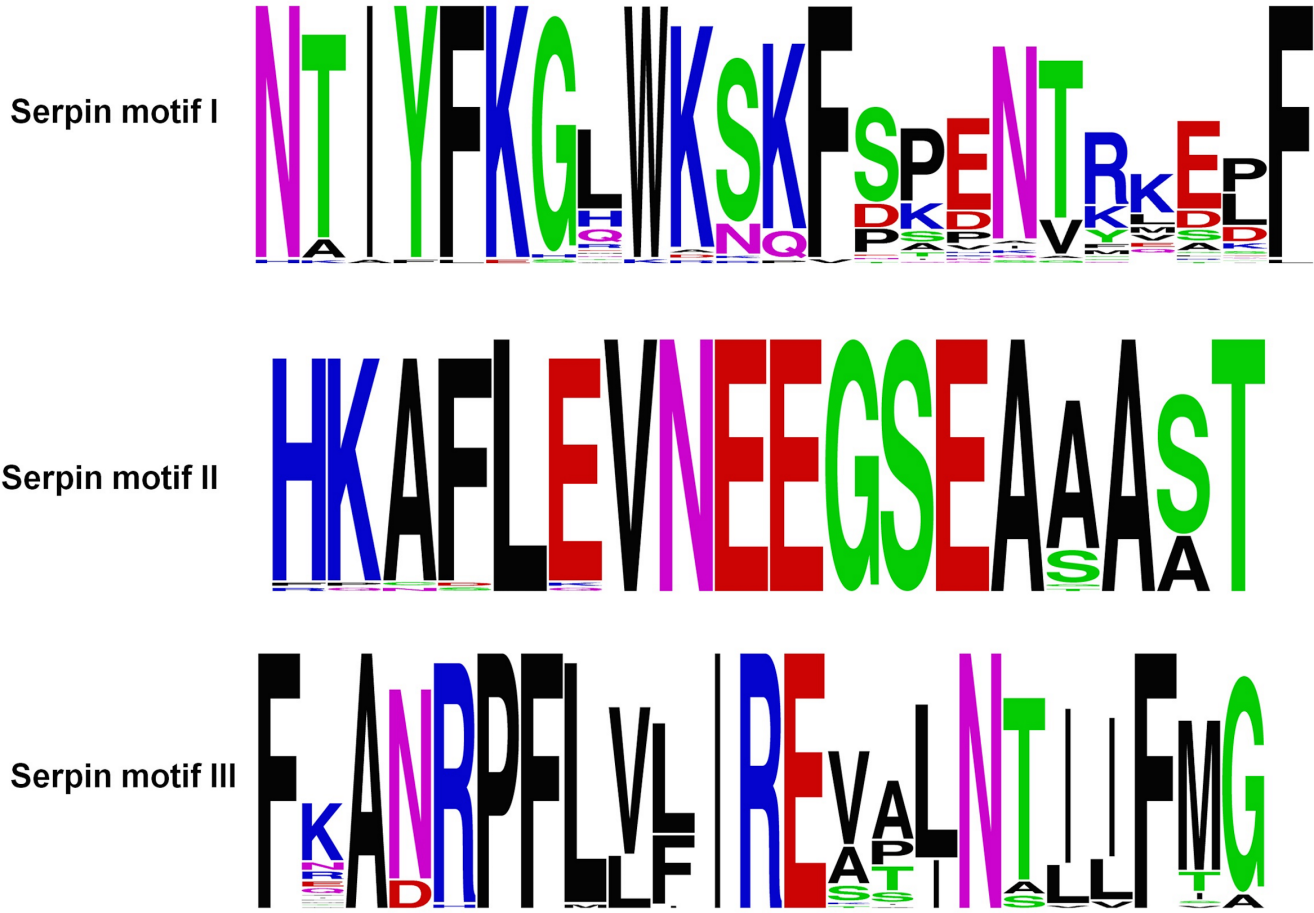
Serpin motifs of ATIII proteins.

**Figure 4:**
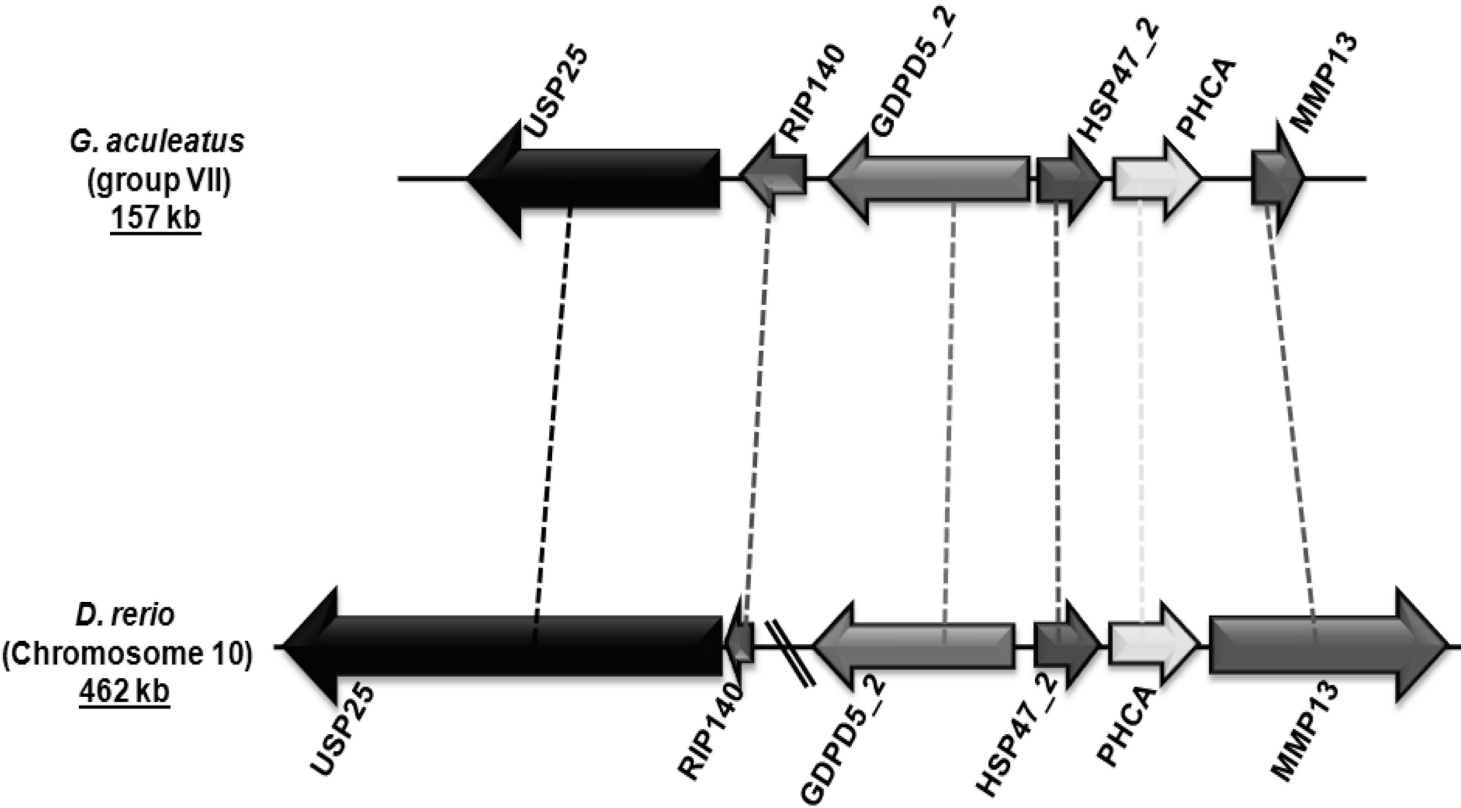
Genomic localization of fish-specific HSP47_2 gene.

Protein sequence analyses depicts that orthologs of PEDF/SERPINF1 and *α*_2_-AP (SERPINF2)-like genes are conserved throughout vertebrates (Kumar, 2010). However, on close scrutiny of group V4 serpins, orthologs of most human group V4 serpins other than A2AP1_FRU in *Fugu* cannot be found in current genomic sequence versions of fish genomes (Kumar, 2010). Fishes have paralogs, probably due to fish-specific genome duplications and diversifications (Kumar, 2010). In addition, the sea lamprey genome has two members of group V4 were detected resembling *α*_2_-AP-like genes (A2APL1_PMA and A2APL2_PMA) with orthologs in the European sea lamprey (Table 1). This suggests that group V4 serpins existed since the beginning of vertebrates. Recently, it was shown that the A2APL1_PMA gene is present in the nested state in the largest intron of PIK4CA gene along with HCII/SERPIND1 gene in the reverse orientation (Kumar et al., 2014a). However, only HCII/SERPIND1 gene is found as nested gene in PIK4CA in the ray-finned fishes to humans (Kumar et al., 2014a) and hence, it is postulated that ancestral group V4 gene were originated at adjacent to HCII/SERPIND1, which was mostly likely lost in the other lineages. This also corroborates that the origin of group V4 serpins is associated with group V2, as assumed from the conservation of basal intron at the position 192a (Fig. 2).

### Group V5 comprises only antithrombin III (ATIII) aka SERPINC1

Group V5 consists of a single member—antithrombin III (ATIII/SERPINC1). This gene encompasses seven exons and six introns with conserved intron positions (Fig. 2). In the human genome, the ATIII/SERPINC1 gene is located on chromosome 1q23–q25. ATIII/SERPINC1 is the major thrombin inhibitor in the blood coagulation cascade (Jordan, 1983), requires heparin for activation and has potent anti-angiogenic activity in certain conformations (Gettins, Patston & Olson, 1996).

The ATIII/SERPINC1 protein is highly conserved, and the sequence identity and similarity from fishes to mammals falls within the range of 50–87% and 67–97% respectively. ATIII/SERPINC1 has been maintained for over 450 MY on the same genomic loci in vertebrates with a few changes in ray-finned fishes. ATIII/SERPINC1 gene has lost an intron (262c) in tetrapods and in the lobed-finned fish coelacanth, *Latimeria chalumnae*. In addition, it has gained an intron at the position 262c in the ray-finned fishes, a characteristic feature, which is shared by group V1 members as well (Kumar et al., 2013a). ATIII/SERPINC1 comprises of several proteins motifs (Fig. 3), heparin binding basic residues, the hD helix, 3 pairs of Cys-Cys salt bridges, *N*-glycosylation sites, serpin motifs and inhibitory RCL (Kumar et al., 2013a). 1997 ATIII/SERPINC1 variants have been identified from 1,092 human genomes. These variants have been categorized into 76.2% SNPs, 11.8% deletions and 8.1% insertions (Kumar et al., 2013a).

### Group V6 is composed of HSP47 (SERPINH1) ortholog and fishes have 1–3 paralogs of HSP47

Group V6 is characterized by a gene structure depicting three introns at positions 192a, 225a and 300c in their coding regions (Fig. 2). This gene encodes for heat shock protein 47 kDa (HSP47/SERPINH1), which possesses a C-terminal endoplasmic reticulum (ER) retention signal (Pelham, 1990). HSP47/SERPINH1 is a non-inhibitory serpin that is found in the ER of collagen producing cells where it is involved in the correct folding of procollagen triplet helices. Furthermore, it assists in the transport of procollagen from the ER to the Golgi complex (Hendershot & Bulleid, 2000; Lamande & Bateman, 1999; Nagata, 1996; Sauk, Nikitakis & Siavash, 2005). Tetrapods have a single copy of HSP47 genes, while fishes have up to three copies. The first HSP47/SERPINH1 is common to all vertebrates (HSP47_1). The second copy is conserved in few ray-finned fishes, such as *G. aculeatus* and *D. rerio* with conserved syntenic organization (Fig. 4). The third one is only present in some fishes such as *D. rerio* (Table 3). HSP47/SERPINH1-like gene is conserved from lampreys to mammals, and this gene show 22–96% sequence identity and 37–98% sequence similarity with human HSP47/SERPINH1, respectively (Table 3). The HSP47_TNI protein is highly diverged from standard HSP47/SERPINH1 protein as well as from all other serpin sequences (Table 3).

**Table 3:** Sequence comparisons of HSP47 homologs in vertebrates. Percentage sequence identity (SI) and percentage sequence similarity (SS) values are shown as compared to HSP47_HSA and A1AT_HSA. Synteny based clustering divides group V6 genes into three sets: set I—true mammalian HSP47 orthologs (normal), set II—fish specific paralogs (bold) and set III (italics).

Human Serpins	Values (%)	HSP47_ MMU	HSP47_ RNO	HSP47_ GGA	HSP47_ XTR	HSP47_ 1_FRU	HSP47_ 2_FRU	HSP47_ TNI	HSP47_ 1_DRE	HSP47_ 2_DRE	HSP47 _3_DRE	HSP47_ PMA
HSP47_HSA	SI	96	96	76	70	**63**	*29*	*22*	65	**64**	*29*	46
	SS	98	98	88	83	**82**	*46*	*37*	83	**82**	*52*	65
A1AT_HSA	SI	23	23	25	24	**24**	*18*	*14*	25	**24**	*17*	23
	SS	45	45	45	46	**44**	*35*	*26*	45	**46**	*37*	41

Orthology of the group V6 gene, lamprey HSP47_PMA (normal font) cannot be decided on this basis. Group V6 comprises of the HSP47 gene and its paralogs in different vertebrates. Tetrapods have a single copy of the HSP47/SERPINH1 gene, while there are two or three HSP47-like genes in some fishes (Fig. 1).

### Status of thrombin inhibitors

Four human serpins inhibit thrombin, namely ATIII/SERPINC1, HCII/SERPIND1, protein C inhibitor (PCI/SERPINA5) and nexin I//SERPINE2 (Huntington, 2013; Huntington, 2014). These serpins exhibit higher rates of thrombin inhibition after binding to the glycosaminoglycan (GAG); however they have evolved radically different inhibition mechanisms (Huntington, 2013). Apart from these four, recent studies revealed that a fifth serpin, namely AGT/SERPINA8 gene also act as thrombin inhibitor,—at least in lampreys (Kumar, Sarde & Bhandari, 2014; Wang & Ragg, 2011; Wong & Takei, 2011). Lamprey AGT/SERPINA8 gene possesses inhibitory RCL and regulates thrombin along with HCII (Kumar, Sarde & Bhandari, 2014). Lampreys have no ATIII/SERPINC1 gene (Kumar et al., 2013a), but after the emergence of ATIII/SERPINC1 gene in tetrapods, AGT/SERPINA8 gene became non-inhibitory serpin (Kumar, Sarde & Bhandari, 2014). It is surprising that lampreys do not have the major thrombin inhibitor, ATIII/SERPINC1. However, lampreys also lack other immunologically critical genes such as recombination-activation genes which are essential for V(D)J recombination process that yields and assembles the variable regions of immunoglobulin and T-cell receptor genes in developing B- and T-lymphocytes (Kumar et al., 2015). This suggests that lampreys may not need some of the very essential vertebrate genes and ATIII/SERPINC1 is in this category. This can be explained since AGT/SERPINA8 is the bi-functional serpin in lampreys, which acts as a thrombin inhibitor as well as a blood pressure regulator (Kumar, Sarde & Bhandari, 2014; Wang & Ragg, 2011; Wong & Takei, 2011).

Major thrombin regulating thrombin serpins are ATIII/SERPINC1 (Kumar et al., 2013a) and HCII/SERPIND1 (Kumar et al., 2014a), which facilitates thrombin inhibition in two different locations such as in the vascular space and in the extravascular space, respectively.

### Special genomic characteristics of serpins

Various types of gene rearrangements characterize a typical evolution of genome. The evolutionary history of serpins is demarked by several such gene rearrangements. Several duplications were the results for expansions of groups V1 (Benarafa & Remold-O’Donnell, 2005) and V2 serpins (Forsyth, Horvath & Coughlin, 2003), from the basal loci of SERPINB1/SERPINB6 and SERPINA8/SERPIND1 in lampreys, respectively. In addition, several serpins in vertebrates are localized as single serpins by chromosomal duplication events such as AGT/SERPINA8 (Kumar, Sarde & Bhandari, 2014), ATIII/SERPINC1 (Kumar et al., 2013a) and HCII/SERPIND1 (Kumar et al., 2014a). HCII/SERPIND1 is conserved from lampreys to humans for ∼500 MY, as a nested gene in the largest intron of PIK4CA gene. This is the only serpin that is known so far, to be nested or overlapped (Kumar et al., 2014a), and was initially reported in *Takifugu* (Kumar, 2009a). Nested gene is a gene that is located within a larger gene (Assis et al., 2008; Kumar, 2009b). There are two types of nested genes, either “within intron” genes, which are nested within the intron of the host gene or “non-intronic” genes, nested within the exonic region of the host gene (Kumar, 2009b).

The most interesting part of genomic characters of the serpins is the changes of exon/intron patterns in the vertebrate serpins via either insertion or deletion of spliceosomal introns. Spliceosomal introns and its splicing machinery are the hallmarks of eukaryotes and this adds subtle intricacies to the gene regulation mechanism. However, formation of congruent sequences by mere chance, execution of effective splicing and maintenance of these sequences for several million years due to certain selective forces, remains quite an enigma (Roy & Gilbert, 2006). Intron invasion is assumed to have happened early on in evolution. Nevertheless, there are several examples of late insertion of introns.

**Figure 5:**
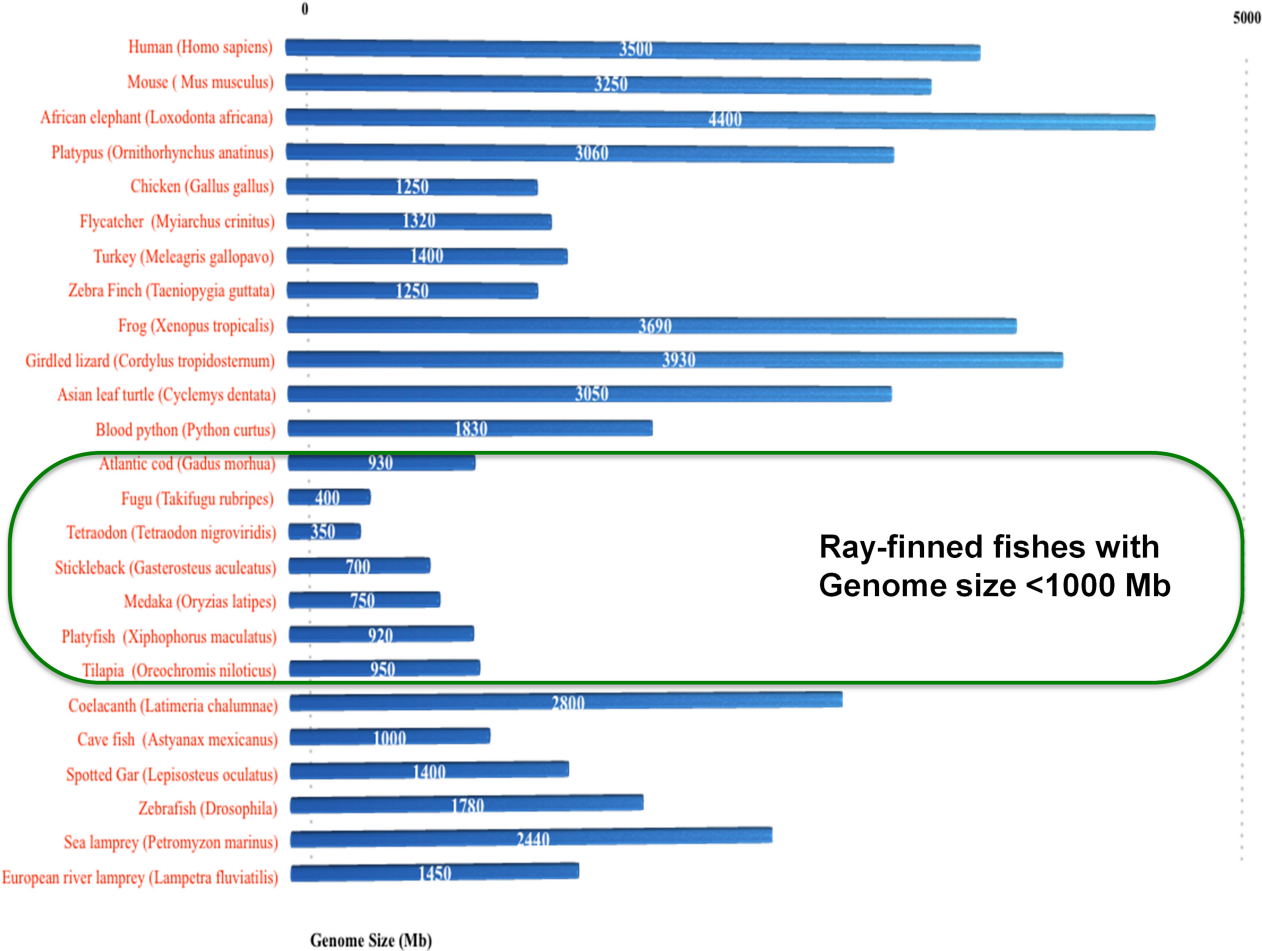
Spliceosomal introns are inserted only in selected ray-finned fishes with genome size lower than 1,000 Mb.

In total, 24 conserved introns are reported in vertebrate serpins encompassing group V1–V6 (Kumar & Ragg, 2008), with six additional introns that were gained in selected ray finned fishes among serpin genes (Ragg et al., 2009). Notably, the intron gains in the non-serpin domain of C1IN have also been reported in these selected fishes (Kumar et al., 2014b). Selected ray-finned fishes (namely *Fugu*, medaka, platyfish, *Tetraodon*, tilapia and stickleback) have novel introns and these fishes have genomes ranging in size from 350–950 Mb or below 1,000 Mb (green box in Fig. 5). Introns are either gained or lost through out eukaryotic evolution (Fedorov et al., 2003; Lee & Chang, 2013; Lin et al., 2006; Roy & Irimia, 2009; Verhelst, Van de Peer & Rouzé, 2013; Yenerall, Krupa & Zhou, 2011; Yenerall & Zhou, 2012; Zhu & Niu, 2013). The only case of intron loss was observed in the ATIII/SERPINC1 gene (Kumar et al., 2013a).

The mechanisms proposed by Yenerall’s group for spliceosomal introns insertions (Yenerall, Krupa & Zhou, 2011; Yenerall & Zhou, 2012) are as follows: (a) intron transposition with partial recombination, (b) transposon insertion, (c) tandem genomic duplication using duplicated splice sites, (d) double-strand break repair (DSBR), (e) group II intron insertion, (f) intron transfer, and (g) intronization. Double-strand break repair (DSBR) coupled with genome compaction events are the driving forces for several examples of intron insertions in selected ray-finned fishes whose genome underwent compaction events in different group of superfamilies such as in the serpin core domain (Ragg et al., 2009), the non-core domain of serpins (Kumar et al., 2014b) and in the selected G-protein coupled receptors (Kumar et al., 2011).

Intron-exon and higher sequence similarities were maintained in serpins of a particular lineage such as in vertebrates (Fig. 2), urochordates (Kumar & Bhandari, 2014) and within insects (Kumar et al., 2014c). With several 1,000 genomes being currently underway, this issue of gene structure pattern will be revisited within the next decade, when several new animal genomes will be available in the databases.

## Conclusions

By utilizing Bayesian phylogenetic method, this report corroborated that the six vertebrate serpins groups are conserved from lampreys to humans for circa 500 MY. Moreover, this study provides several vignettes of vertebrate serpins from genomic and phylogenetic perspectives.

## Supplemental Information

10.7717/peerj.1026/supp-1 Table S1 Maximum Likelihood fits of *50* different amino acid substitution models of alignment of serpins using MEGA 5 The lowest BIC scores (Bayesian Information Criterion) are considered for the best fit of the substitution pattern.
